# Effect of Cold Deformation on the Microstructural and Property Uniformity of Al_2_O_3_/Cu Composites

**DOI:** 10.3390/ma18010125

**Published:** 2024-12-31

**Authors:** Song Liu, Shaolin Li, Kexing Song, Xiuhua Guo, Hao Song, Keke Qi, Fuxiao Chen

**Affiliations:** 1 School of Materials Science and Engineering, Henan University of Science and Technology, Luoyang 471023, China; asi-911@163.com (S.L.); lsl0379@haust.edu.cn (S.L.); guoxiuhua@haust.edu.cn (X.G.); 220320020178@huast.edu.cn (H.S.); qikele118@163.com (K.Q.); 2 School of Mechanical and Aeronautical Manufacturing Engineering, Anyang Institute of Technology, Anyang 455000, China; 3 Collaborative Innovation Center of Nonferrous Metals, Luoyang 471023, China; 4 Key Laboratory of Materials Science and Processing Technology for Non-Ferrous Metals of Henan, Luoyang 471023, China; 5 Henan Academy of Sciences, Zhengzhou 450002, China; 6 Henan Provincial Key Laboratory of Advanced Conductor Materials, Zhengzhou 450046, China

**Keywords:** Al_2_O_3_/Cu composite, cold rolling deformation, mechanical property, finite element analysis, dislocation strengthening

## Abstract

Copper matrix composites (Cu-MCs) have garnered significant attention due to their exceptional electrical, wear-resistant, and mechanical properties. Among them, Al_2_O_3_/Cu composites, reinforced with Al_2_O_3_, are a focal point in the field of high-strength, high-conductivity copper alloys, owing to their high strength, excellent electrical conductivity, and superior resistance to high-temperature softening. Cold deformation is an effective method for enhancing the mechanical properties of Al_2_O_3_/Cu composites. However, during cold deformation of large-cross-sectional Al_2_O_3_/Cu composites, the inhomogeneity in microstructure and properties induced by varying stress states cannot be overlooked. In this study, cold deformation of 1.12 wt% Al_2_O_3_/Cu large-cross-sectional composites was performed using a rolling process, coupled with finite element numerical simulations, to investigate the distribution characteristics of microstructure and properties during the rolling process. The results indicate that under cold deformation, the hardness of the material increases linearly from the surface layer to the core, while the change in electrical conductivity is minimal. The increase in hardness is closely related to variations in dislocation density and grain size, with dislocation density being the dominant strengthening mechanism. Quantitative analysis reveals that strain inhomogeneity during cold deformation is the primary cause of microstructural differences, leading to variations in mechanical properties at different positions. This study provides a theoretical basis for understanding the inhomogeneity of cold deformation in large-sized Al_2_O_3_/Cu composites and for controlling their microstructure–property relationships.

## 1. Introduction

Al_2_O_3_/Cu composites are well known for their high strength, excellent electrical conductivity, superior wear resistance, and exceptional resistance to high-temperature softening, making them key materials in developing high-strength and high-conductivity copper alloys. These materials have become a central research focus in high-strength, high-conductivity copper alloys [1] and are widely applied in critical sectors such as rail transportation, national defense, and nuclear reactors [2].

Al_2_O_3_/Cu composites are copper alloys that incorporate Al_2_O_3_ particles, known for their high melting points, hardness, excellent thermal stability, and chemical inertness, into copper-based alloys through in situ or ex situ methods [3]. The most commonly used techniques for preparing Al_2_O_3_/Cu composites are in situ and ex situ synthesis [4]. In situ synthesis involves reactions within the metal matrix to generate the reinforcing phase, such as internal oxidation and spark plasma sintering, while ex situ synthesis directly adds reinforcing particles to the matrix, such as mechanical alloying and powder metallurgy. Among these methods, Al_2_O_3_/Cu composites produced via internal oxidation demonstrate superior wettability between Al_2_O_3_ particles and the copper matrix, resulting in improved overall properties. This method has become the preferred industrial process for manufacturing Al_2_O_3_/Cu composites [5]. The typical internal oxidation process includes mixing oxidizing agents with Cu–Al alloy powder, isostatic pressing to form a billet, conducting internal oxidation under controlled atmospheric conditions, and performing hot extrusion to produce rods. Research indicates that increasing the Al_2_O_3_ content improves the mechanical properties and high-temperature stability of Al_2_O_3_/Cu composites. However, higher Al_2_O_3_ content results in a decrease in electrical conductivity and plasticity. Ren et al. [6] prepared 0.32 wt% Al_2_O_3_/Cu composites through internal oxidation, achieving excellent electrical conductivity of 94.1% IACS, but a relatively low tensile strength of 445 MPa. Lee et al. [7] fabricated 0.94 wt% Al_2_O_3_/Cu composites via internal oxidation, achieving high tensile strength (565 MPa), but with a significant reduction in electrical conductivity (77.4% IACS). Therefore, maintaining high mechanical properties while preserving electrical conductivity is critical for the practical application of Al_2_O_3_/Cu composites. Studies have shown [8,9] that cold deformation techniques are essential for enhancing the mechanical properties of metal materials while maintaining electrical conductivity. Peng et al. [10] applied significant cold drawing deformation to annealed 0.2 wt% Al_2_O_3_/Cu composites, resulting in elongated fibrous structures that increased microhardness, yield strength, and tensile strength with minimal impact on conductivity. Zhang et al. [3] performed cold drawing on 1.12 wt% Al_2_O_3_/Cu composites, and the results indicated that increased deformation led to higher density and dislocation density, resulting in significant improvements in hardness and strength, while conductivity remained largely unchanged. Thus, cold deformation offers a promising approach to improving the mechanical properties of Al_2_O_3_/Cu composites while preserving their electrical conductivity. However, current studies on the cold rolling of Al_2_O_3_/Cu composites are predominantly focused on plates and rods with diameters smaller than 20 mm. Research on the cold rolling process of large-cross-section Al_2_O_3_/Cu composite rods remains scarce [11]. To address this gap, this study employs an internal oxidation process to prepare a large-cross-section 1.12 wt% Al_2_O_3_/Cu composite rod with dimensions of 46 mm × 35 mm. The research systematically investigates the microstructural evolution along the rolling direction during cold rolling, analyzing the effects of deformation on the mechanical properties and electrical conductivity at different positions within the cross-section of the large-cross-section Al_2_O_3_/Cu composite rod. Furthermore, finite element numerical simulation software (DEFORM v11.0) is utilized to simulate the rolling process of the composite material. This study establishes the relationship between the deformation behavior, microstructural evolution, and property inhomogeneity along the rolling direction of the cross-section. Additionally, the strengthening mechanisms at different positions within the large-cross-section 1.12 wt% Al_2_O_3_/Cu composite rod and their respective contributions are explored. The findings of this study provide valuable theoretical insights for optimizing the forming processes of large-cross-section Al_2_O_3_/Cu composite rods and improving their mechanical properties.

## 2. Experimental Procedure

### 2.1. Raw Materials

**Figure 1:**
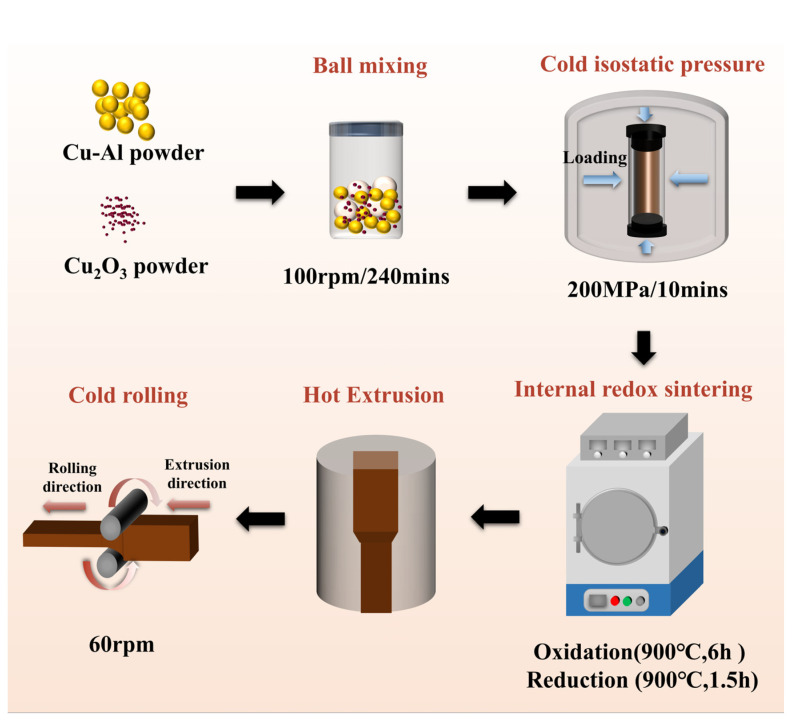
Schematic diagram of the preparation process for 1.12 wt% Al_2_O_3_/Cu composite, including powder mixing, cold isostatic pressing, internal oxidation–reduction–sintering, hot extrusion, and cold rolling.

The Cu–0.6 wt% Al alloy powder (average particle size: 39 μm, purity: 99.7%, purchased from Hunan Huabang Powder Materials Co., Ltd., Liuyang, Hunan Province, China) and Cu_2_O powder (average particle size: 1 μm, purity: 99.9%, purchased from Shanghai Xiangtian Nanomaterials Co., Ltd., Fengxian District, Shanghai, China) were used as raw materials. These powders were uniformly mixed in a powder mixing machine at a rotational speed of 100 rpm for 240 min to ensure homogeneity. The mixed powders were then subjected to cold isostatic pressing (CIP) at a pressure of 200 MPa for 10 min to form a compacted billet. This step was essential to ensure uniform density and structural integrity before sintering, as supported by the relevant literature [12]. The internal oxidation–reduction–sintering process was performed in an SG-1XQL vacuum atmosphere furnace. Internal oxidation was conducted at 900 °C for 6 h under an argon atmosphere. The reduction process was subsequently performed at 900 °C for 1.5 h in a hydrogen–argon mixed gas atmosphere. These steps ensured complete internal oxidation and reduction of the billet, followed by furnace cooling. The compacted and sintered billet was then subjected to hot extrusion using a YLX horizontal four-column hydraulic press to achieve densification. The extruded rod had cross-sectional dimensions of 46 mm × 35 mm. Cold rolling deformation was carried out along the extrusion direction in two passes, with a reduction of 5 mm per pass, resulting in a total deformation ratio of approximately 20%. After cold rolling, the final cross-sectional dimensions of the rod were reduced to 36 mm × 38 mm. To enhance clarity and readability, the experimental process is summarized in Figure 1, which visually illustrates each step, including powder mixing, cold isostatic pressing, internal oxidation–reduction–sintering, hot extrusion, and cold rolling.

### 2.2. Characterization

Electrical conductivity was measured using the Sigma 2008B1 eddy current conductivity meter (Xiamen Tianyan Instruments Co., Ltd., Torch High-tech Industrial Development Zone, Xiamen, China). Measurements were conducted at multiple positions on the sample, with five tests performed at each position and the average value recorded. Hardness was measured using the HBST-3000 digital Brinell hardness tester, with a 2.5 mm diameter carbide steel ball indenter, applying a load of 180 kgf for 30 s. Each sample was tested five times, and the average value was used. Scanning electron microscopy (SEM, JSM-IT100, JEOL, Tokyo, Japan) was employed to observe the microstructural evolution after cold rolling. The grain size was analyzed using ImageJ 1.54d software and the line-intercept method, with at least 200 grains counted per micrograph to ensure accuracy. X-ray diffraction was performed using a Bruker D8 X-ray diffractometer (Karlsruhe, Germany). Transmission electron microscopy (TEM, JSM-2100, JEOL) was employed to observe the characteristics of Al_2_O_3_ particles and grains, with data processing carried out using Digital Micrograph 3 software.

### 2.3. Finite Element Simulation

In the finite element model, the cross-sectional dimensions of the rolled rod are 46 mm × 35 mm. The model is meshed with a total of 524,941 tetrahedral elements. To prevent mesh distortion during large plastic deformations, the automatic re-meshing function is employed to maintain adequate capturing of the deformation region, which aids in the accurate calculation and analysis of key parameters such as stress and strain during the deformation process. The geometric diameter of the rolls is 500 mm, with a rotational speed of 60 rpm and a reduction of 20%. The rolls are simplified as analytical rigid bodies [13]. The Al_2_O_3_/Cu composite is modeled as a plastic material, and the flow stress constitutive equation is automatically calculated by DEFORM based on tensile test data. Coulomb friction is applied, with a friction coefficient of 0.3, and the thermal conductivity is set to 322 W/(m·K).

## 3. Results and Discussion

### 3.1. Effect of Cold Deformation on the Microstructure of Al_2_O_3_/Cu Composites

**Figure 2:**
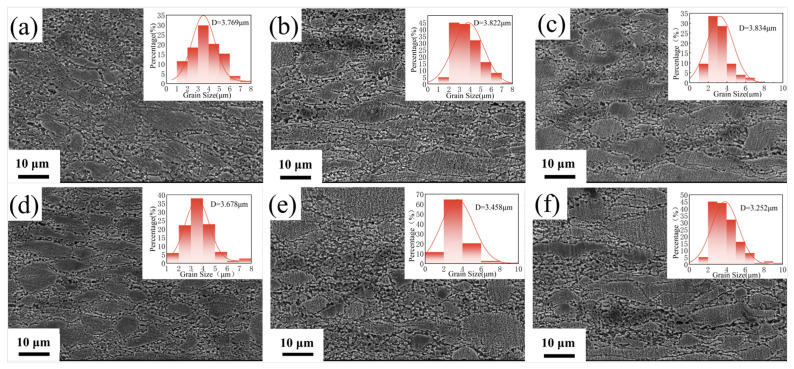
Scanning electron microscopy (SEM) images and grain size distribution of Al_2_O_3_/Cu composites in the cross-section along the rolling direction: (**a**) center layer before rolling; (**b**) 1/4 layer before rolling; (**c**) surface layer before rolling; (**d**) center layer after rolling; (**e**) 1/4 layer after rolling; (**f**) surface layer after rolling.

Figure 2 illustrates the scanning electron microscope (SEM) images and grain size distribution maps of Al_2_O_3_/Cu composites at different positions along the rolling cross-section, both before and after rolling. Figure 2a–c show the center layer, 1/4 layer, and surface layer of the billet before rolling, while Figure 2d–f depict the corresponding layers after rolling. A comparison of the grain size distribution maps (Figure 2a–f) indicates that after rolling, the average Cu grain size decreases, with varying reductions across different positions. The surface layer experiences the most significant decrease. Before rolling, the Cu grains are relatively large, with average sizes ranging from 3.769 to 3.834 μm, and are fairly uniform. After rolling, the Cu grains are noticeably refined, with the extent of refinement varying across positions. Specifically, the surface layer’s grain size decreases from 3.834 μm to 3.252 μm, while the core only decreases from 3.769 μm to 3.678 μm.

**Figure 3:**
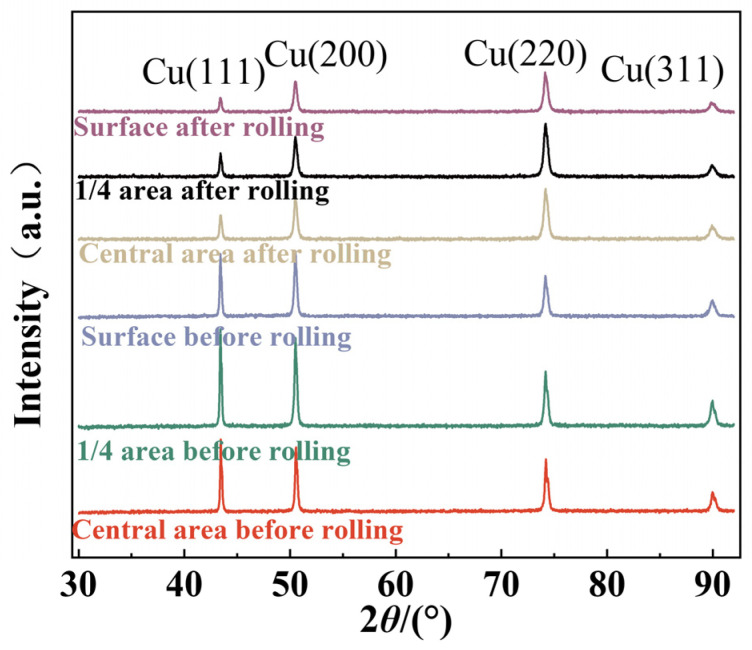
X-ray diffraction (XRD) patterns of Al_2_O_3_/Cu composites along the rolling direction in the cross-section before and after cold rolling.

Figure 3 presents the X-ray diffraction (XRD) patterns of Al_2_O_3_/Cu composites at different positions along the rolling direction in the cross-section of the rolled specimen both before and after rolling. Due to the low content of Al_2_O_3_ particles, no diffraction peaks for Al_2_O_3_ are observed in the XRD patterns. After cold rolling, significant changes in the intensity of the main diffraction peaks are observed. In the undeformed state, the copper single-crystal orientation is primarily concentrated at the (111) and (200) planes. During cold deformation, dislocation slip causes changes in the crystal orientation, eventually stabilizing at the (220) crystal plane. The increase in residual stress within the material leads to the transition from single to dual, multiple, and cross-slip systems, resulting in substantial work hardening. For crystal rotation, the most stable configuration occurs when the crystal plane is aligned with the rolling direction. Under shear stress, the (111) and (200) crystal planes continuously rotate toward the (220) plane. Although the deformation is minor, the grain orientation gradually aligns with the rolling direction. Similarly, when studying the effects of different deformation amounts on the microstructure, electrical conductivity, and mechanical properties of Al_2_O_3_/Cu composites during cold rolling, Song Hao et al. [12] observed that the (111) and (200) crystal planes continuously decreased, ultimately rotating to the (220) plane, with the grain orientation progressively aligning along the rolling direction.

### 3.2. Effect of Cold Deformation on the Properties of Al_2_O_3_/Cu Composites

**Figure 4:**
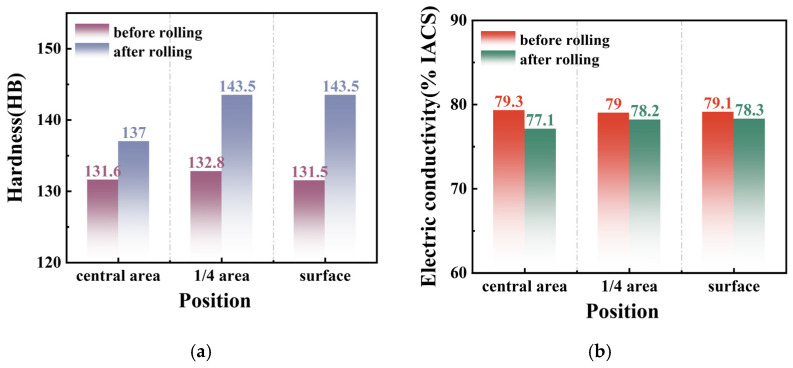
Variation in hardness and electrical conductivity before and after cold rolling at different positions along the rolling direction in the cross-section of Al_2_O_3_/Cu composites: (**a**) hardness variation; (**b**) electrical conductivity variation.

Figure 4 illustrates the variation in electrical conductivity and hardness at different positions along the rolling direction in the cross-section of Al_2_O_3_/Cu composites before and after cold rolling deformation. As rolling progresses, hardness shows a significant change, whereas electrical conductivity remains relatively stable. The hardness increases substantially from approximately 132 HB in the initial state to around 140 HB, indicating considerable work hardening in the composite. In contrast, electrical conductivity decreases slightly from 79% IACS to 78% IACS, representing a modest reduction of only 1%. This change is attributed to the increased dislocation density and grain refinement, which enhance electron scattering; however, this effect has a negligible impact on electrical conductivity in Al_2_O_3_/Cu composites.

### 3.3. Finite Element Simulation Analysis

**Figure 5:**
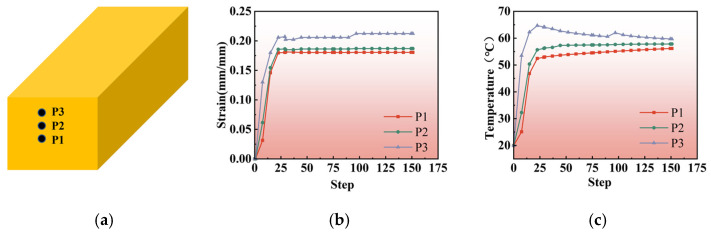
Numerical simulation results for points P1, P2, and P3 at different locations along the rolling direction in the cross-section: (**a**) locations of points: P1—center layer, P2—quarter layer, P3—outer layer; (**b**) strain variation curves for points P1, P2, and P3 at different process steps; (**c**) temperature variation curves for points P1, P2, and P3 at different process steps.

**Figure 6:**
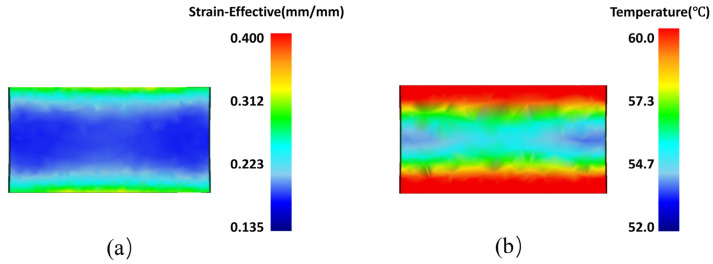
Cross-sectional contour plots along the rolling direction: (**a**) strain contour plot; (**b**) temperature contour plot.

The microstructural changes and mechanical property inhomogeneities of Al_2_O_3_/Cu composites are attributed to billet deformation during the rolling process. The degree of material deformation is commonly assessed based on the equivalent strain within the material [13]. Finite element methods were utilized to numerically simulate the strain distribution during the rolling process, as illustrated in Figure 5 and Figure 6. When the billet cross-sectional area is large along the rolling direction, deformation varies across different positions in the cross-section. Therefore, strain and temperature variations were tracked at three specific points during each pass: the center layer (P1), the 1/4 layer (P2), and the surface layer (P3), as depicted in Figure 5a. The corresponding strain and temperature curves were then traced for these points.

During the rolling process, the composite material’s surface layer interacts with the rolls, moving forward due to the frictional force, thereby driving the 1/4 and center layers. Figure 5b,c illustrate the variations in equivalent strain and temperature at the center (P1), 1/4 layer (P2), and surface (P3) points along the cross-section of the rolled specimen. As shown in Figure 5 and Figure 6, after engagement with the rolls, the strain increases rapidly in all layers. The surface layer undergoes the highest strain, while the center layer experiences the lowest. The strain difference between the layers grows as deformation progresses, and the heat generated during deformation raises the billet’s temperature from the inside out.

During the rolling process of the composite material, the stress–strain relationship can be effectively modified by adjusting and optimizing the reduction amount in each pass, deformation parameters, and friction conditions between the rolls and the workpiece. This approach alleviates deformation inhomogeneity and performance variations during rolling [14].

### 3.4. Strengthening Mechanisms and Strength Contributions of Al_2_O_3_/Cu Composites

**Figure 7:**
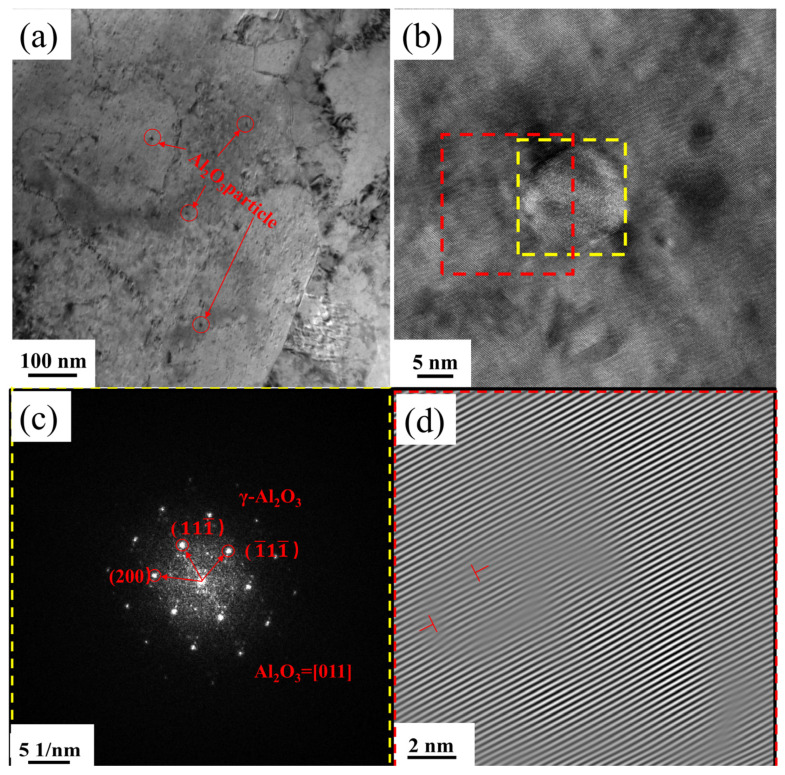
Transmission electron microscopy (TEM) images of Al_2_O_3_/Cu composites: (**a**,**b**) microstructural morphology showing the distribution of Al_2_O_3_ particles; (**c**) fast Fourier transform (FFT) image of the yellow region in (**b**); (**d**) inverse fast Fourier transform (IFFT) image of the red region in (**b**).

Figure 7 presents the TEM images of Al_2_O_3_/Cu composites. In Figure 7a, the Al_2_O_3_ particles formed by internal oxidation are clearly visible, displaying nanoscale sizes and a uniform, dispersed distribution within the copper matrix without the presence of large particles. As shown in Figure 7c, the shaded particles in Figure 7b are confirmed to be in-situ-formed γ-Al_2_O_3_ particles, with a diameter of approximately 16 nm. Figure 7d reveals dislocations around the Al_2_O_3_ particles. During cold rolling deformation, plastic deformation in the composite occurs primarily through planar slip. The improvement in the mechanical properties of the composite after cold deformation is attributed to the interaction between the reinforcing phase particles and dislocations.

Extensive studies have investigated the strengthening mechanisms of copper-based composites, focusing primarily on grain boundary strengthening, Orowan strengthening, dislocation strengthening, solid solution strengthening, and load transfer strengthening [15,16]. The strengthening of Al_2_O_3_/Cu composites is generally influenced by the extent of deformation. Specifically, the tensile strength of metal materials is predominantly attributed to grain boundary strengthening, solid solution strengthening, dislocation strengthening, precipitation strengthening, and Orowan strengthening [17,18]. Due to the nanoscale size of the Al_2_O_3_ particles dispersed in copper, the strengthening during cold rolling mainly results from grain boundary strengthening, Orowan strengthening due to the pinning effect of Al_2_O_3_ particles within the alloy, and dislocation strengthening. The tensile strength of Al_2_O_3_/Cu composites under various deformation conditions can be estimated using the following equation:

(1) $$\sigma_{\mathrm{TS}}=\sigma_{m}+\Delta \sigma_{\mathrm{gb}}+\Delta \sigma_{\mathrm{OS}}+\Delta \sigma_{\mathrm{dis}}$$

where *σ*_TS_ represents the tensile strength of the Al_2_O_3_/Cu composite, *σ*_m_ is the tensile strength of the copper matrix, Δ*σ*_gb_ denotes grain boundary strengthening, Δ*σ*_OS_ represents Orowan strengthening induced by nanoscale Al_2_O_3_ particles, and Δσ_dis_ refers to dislocation strengthening caused by these particles.

Grain boundary strengthening can be calculated using the Hall–Petch equation [19]:

(2) $$\Delta \sigma \mathrm{gb}=\sigma_{0}+\frac{K}{\sqrt{d}}$$

In the equation, *σ*_0_ represents the friction stress of copper (75 MPa) [20], *K* is the Hall–Petch coefficient with a value of 4.5 MPa·mm^−2^, and d is the average grain size. According to the Hall–Petch equation, the grain boundary strengthening (Δ*σ*_gb_) depends on the friction stress of copper, the Hall–Petch coefficient *K*, and the grain size d. Due to uneven deformation during rolling, the grain size d varies at different positions in the cross-section of large-diameter dispersion-strengthened copper rods. For a given volume fraction of Al_2_O_3_ particles, smaller grain sizes d result in stronger grain boundary strengthening and higher Δ*σ*_gb_ values. As shown in Figure 1, the rolled billet exhibits the smallest grain size at the surface layer and the largest in the core. Therefore, the grain boundary strengthening effect is most pronounced in the surface layer and least in the core. By substituting the known data into the equation, the contributions of grain boundary strengthening to the composite strength are calculated to be 148 MPa, 149 MPa, 152 MPa, and 154 MPa for the surface layer, 1/4 layer, and core before and after rolling, respectively.

The Orowan strengthening induced by nanoscale Al_2_O_3_ particles can be calculated using the following equation [21,22,23]:

(3) $$\Delta \sigma_{\mathrm{OS}}=\frac{0.4Gb}{\pi \sqrt{1-\nu }}\frac{1}{L}\ln\left(\frac{D}{b}\right)$$

In the equation, *G* represents the shear modulus (42.1 GPa), *b* denotes the Burgers vector (0.256 nm), *μ* is the Poisson’s ratio (0.326), *D* indicates the average particle size of Al_2_O_3_, and *L* refers to the interparticle spacing of Al_2_O_3_ particles. The value of *L* can be derived from the particle volume fraction and their average size using the following formula:

(4) $$L=D\left(\sqrt{\frac{\pi }{6V}}-\frac{\pi }{4}\right)$$

From Equations (3) and (4), it is evident that the Orowan strengthening (Δ*σ*_OS_) induced by nanoscale Al_2_O_3_ particles depends solely on the shear modulus of the copper matrix (*G*), the Burgers vector (*b*), the Poisson’s ratio (*μ*), and the average particle size (*D*) and spacing (*L*) of the Al_2_O_3_ particles. Hence, the position in the cross-section does not influence the degree of Orowan strengthening. For this material, with an Al_2_O_3_ volume fraction of 2.2%, substituting the known parameters into the equations yields an Orowan strengthening contribution of 112 MPa at the surface layer, 1/4 layer, and core, both before and after rolling.

Dislocation strengthening is calculated using the Taylor equation [24]:

(5) $$\Delta \sigma \mathrm{dis}=M\alpha Gb\sqrt{\rho }$$

In the equation, M represents the Taylor factor for copper, with a value of 3.06; *α* denotes the dislocation interaction coefficient, which is 0.2 for copper; *G* is the shear modulus (47.7 GPa); *b* is the Burgers vector of copper (0.256 nm); and *ρ* is the dislocation density. The dislocation density (*ρ*) is calculated using the following equation [25]:

(6) $$\beta \cos\theta =\frac{0.9\lambda }{d}+\varepsilon \sin\theta$$

(7) $$\rho =16.1{\left(\frac{\varepsilon }{b}\right)}^{2}$$

In the equation, *β* represents the full width at half maximum (FWHM) of the corresponding Cu diffraction peak at the Bragg angle 2*θ*, and *λ* is the wavelength of the X-ray (0.154 nm). d, *ε*, and *ρ* denote the average grain size, internal elastic strain, and dislocation density of the Cu matrix, respectively, while *b* is the Burgers vector of copper (0.256 nm). The FWHM of the diffraction peak is obtained by performing multi-peak fitting of the XRD spectrum using Origin 2019b software, where Gaussian fitting is applied until convergence to determine the FWHM. The average grain size of the Cu matrix is measured from microstructural images using ImageJ 1.54d software. The internal elastic strain (*ε*) is calculated using Equation (6), and the dislocation density (*ρ*) is determined using Equation (7).

From Equations (5) to (7), it can be observed that dislocation strengthening (Δ*σ*_dis_) is related to the dislocation density (*ρ*). A higher dislocation density (*ρ*) results in a greater dislocation strengthening (Δ*σ*_dis_). The dislocation density (*ρ*) is influenced by the average grain size (d) of the Cu matrix, the Burgers vector (*b*), and the internal elastic strain (*ε*). Larger average grain sizes (d) lead to lower dislocation densities (*ρ*), while higher internal elastic strains (*ε*) result in higher dislocation densities. According to Equation (7), the internal elastic strain (*ε*) is determined by the FWHM (*β*) of the corresponding Cu diffraction peak at the Bragg angle (2*θ*), the X-ray wavelength (*λ*), and the average grain size (d) of the Cu matrix. Larger FWHM values (*β*) correspond to smaller internal elastic strains (*ε*), while smaller average grain sizes (d) also reduce the internal elastic strain (*ε*).

Using the known data and substituting into the equations, the dislocation densities (*ρ*) in the surface layer, 1/4 layer, and core of the billet before rolling are calculated to be 12.5 × 10^14^, 12.1 × 10^14^, and 15.3 × 10^14^ m^−2^, respectively. After rolling, the dislocation densities in the surface layer, 1/4 layer, and core are 16.0 × 10^14^, 22.3 × 10^14^, and 24.6 × 10^14^ m^−2^, respectively. The contributions of the dislocation strengthening mechanism to the composite strength are calculated to be 265 MPa, 260 MPa, and 293 MPa before rolling, and 299 MPa, 353 MPa, and 370 MPa after rolling.

**Figure 8:**
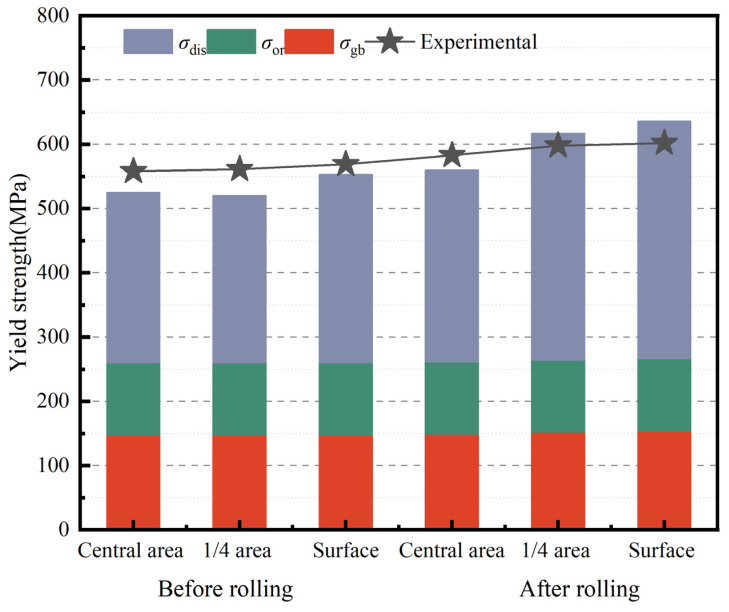
Percentage contribution of strengthening mechanisms to the overall strength of Al_2_O_3_/Cu composites at different cross-sectional positions along the rolling direction, before and after cold rolling.

Figure 8 illustrates the contributions of different strengthening mechanisms to the strength of Al_2_O_3_/Cu composites at various positions along the cross-section before and after rolling. Before rolling, grain boundary strengthening, Orowan strengthening, and dislocation strengthening are the dominant mechanisms. As cold rolling progresses, the contributions of grain boundary strengthening and dislocation strengthening increase significantly. 

Table 1 presents the contributions of these strengthening mechanisms to the tensile strength of the composites at different positions along the same cross-section before and after rolling. The theoretical values and experimental measurements show an error of less than 8.0%, indicating good agreement. After rolling, the average grain boundary strengthening (151 MPa) slightly increases compared to the before rolling value (148 MPa), while the average dislocation strengthening (341 MPa) rises by 25% (69 MPa) from its pre-rolling value (272 MPa). This improvement is attributed to reduced grain size and increased dislocation density during cold deformation.

In summary, during the cold rolling process, the strengthening mechanisms of the composite material primarily include grain boundary strengthening, Orowan strengthening, and dislocation strengthening. The degree of deformation varies across different positions within the cross-section of the billet, leading to distinct distributions of grain size and dislocation density. Notably, Orowan strengthening remains relatively unchanged with increasing deformation, while grain boundary strengthening and dislocation strengthening intensify as deformation progresses. These mechanisms work synergistically to enhance the overall strength of the composite. This aligns with the findings of Orolínová et al. [26], who identified grain boundary and Orowan strengthening as the primary mechanisms governing the room-temperature yield strength of Al_2_O_3_/Cu composites. Wang et al. [27] also highlighted the synergistic strengthening effect in Al_2_O_3_/Cu composites, where Al_2_O_3_ particle dispersion and copper matrix grain refinement contribute together to the overall enhancement of material strength. In the surface layer of the billet, where deformation is most pronounced, the strengthening effect is the most significant. This observation is consistent with Liao et al. [28], who found that cold rolling of C70250 copper alloy strips generated a large number of dislocations within the grains, leading to improved tensile strength. Similarly, Sun et al. [29] showed that dislocations in Cu–Ni–Cr alloys increase rapidly with increasing rolling strain, resulting in enhanced strength due to dislocation strengthening. Compared to the core, the contributions of grain boundary and dislocation strengthening are more pronounced in the surface layer, further emphasizing the influence of deformation on the strengthening mechanisms of the composite.

## 4. Conclusions

In summary, this study not only reveals the hardening mechanisms during cold rolling of large-cross-section Al_2_O_3_/Cu composites but also provides theoretical insights for optimizing the forming process and significantly enhancing their mechanical properties:

1. During cold rolling deformation, the dislocation density increases while the grain size decreases. The billet surface layer undergoes the most pronounced deformation compared to the core, with the dislocation density increasing from 15.3 × 10^14^ m^−2^ before rolling to 24.6 × 10^14^ m^−2^ after rolling, and the grain size decreasing from 3.834 μm to 3.252 μm.
2. After rolling, the hardness of the material exhibits a significant increase, rising from approximately 132 HB in the initial state to around 140 HB, while the electrical conductivity decreases slightly from 79% IACS to 78% IACS. These changes are primarily attributed to the increased dislocation density and refined grain size, which enhance electron scattering. However, this effect has a minimal impact on the electrical conductivity of copper.
3. Finite element numerical simulations established the relationship between deformation patterns and the microstructural and property inhomogeneities across the cross-section of the large-cross-section 1.12 wt% Al_2_O_3_/Cu composite rod during rolling. The strain values are highest in the surface layer and lowest in the core, with the differences between the two increasing progressively throughout the deformation process.
4. The strengthening mechanisms of Al_2_O_3_/Cu composites are closely associated with cold rolling deformation. In the as-received state, the primary mechanisms include Orowan strengthening, grain boundary strengthening, and dislocation strengthening. After rolling, the contributions of grain boundary strengthening and dislocation strengthening to the overall strength increase significantly.

Despite the significant findings, this study has certain limitations, such as experimental conditions, the limited composition of the materials studied, and the simplified finite element modeling. These factors may affect the comprehensiveness and accuracy of the results. Future research will focus on multi-scale simulations, composite design, process optimization, and performance prediction to deepen understanding of cold rolling mechanisms in large-cross-section Al_2_O_3_/Cu composites and improve material performance.

## Figures and Tables

**Table 1:** Contributions of different strengthening mechanisms to tensile strength of Al_2_O_3_/Cu Composites at various cross-sectional positions before and after cold rolling.

Point	*ρ*/ 10^14^ m^−2^	*σ*_gb_/ MPa	*σ*_or_/ MPa	*σ*_dis_/ MPa	Theoretical Value/MPa	Measured Value/MPa	Deviation/%
A1	12.5	148	112	265	525	558	6.3
A2	12.1	148	112	260	520	561	7.8
A3	15.3	148	112	293	553	569	2.9
B1	16.0	149	112	299	560	583	3.9
B2	22.3	152	112	353	617	598	3.2
B3	24.6	154	112	370	636	602	5.6

Notes: *ρ* is the dislocation density, *σ*_gb_ is the grain boundary strengthening, *σ*_or_ is the Orowan strengthening, and *σ*_dis_ is the dislocation strengthening.

## Data Availability

The original contributions presented in the study are included in the article; further inquiries can be directed to the corresponding author.
